# Early Histone Deacetylase Inhibition Mitigates Ischemia/Reperfusion Brain Injury by Reducing Microglia Activation and Modulating Their Phenotype

**DOI:** 10.3389/fneur.2019.00893

**Published:** 2019-08-20

**Authors:** Shuyuan Li, Xiaoshuang Lu, Qian Shao, Zixin Chen, Qiong Huang, Zinan Jiao, Xiaodi Huang, Maosong Yue, Jingwen Peng, Xin Zhou, Dachong Chao, Heng Zhao, Juling Ji, Yuhua Ji, Qiuhong Ji

**Affiliations:** ^1^College of Life Science and Technology, Institute of Immunology, Jinan University, Guangzhou, China; ^2^Key Laboratory of Neuroregeneration, Nantong University, Nantong, China; ^3^Department of Neurology, Affiliated Hospital of Nantong University, Nantong, China; ^4^Department of Pathology, Medical School of Nantong University, Nantong, China

**Keywords:** acute brain ischemia, HDACi, SAHA, inflammation, microglia/macrophages, polarization

## Abstract

Histone deacetylase inhibitors (HDACi) are a promising therapeutic intervention for stroke. The involvement of the anti-inflammatory effects of HDACi in their neuroprotection has been reported, but the underlying mechanisms are still uncertain. Given the post-stroke inflammation is a time-dependent process, starting with acute and intense inflammation, and followed by a prolonged and mild one, we proposed whether target the early inflammatory response could achieve the neuroprotection of HDACi? To test this hypothesis, a single dose of suberoylanilide hydroxamic acid (SAHA) (50 mg/kg), a pan HDACi, was intraperitoneally (i.p.) injected immediately or 12 h after ischemia onset in a transient middle cerebral artery occlusion (tMCAO) mouse model. Compared with delayed injection, immediate SAHA treatment provided more protection, evidenced by smaller infarction volume, and a better outcome. This protection was accompanied by suppression of pro-inflammatory cytokines and reduction of activated microglia in the early stage of post-stroke inflammation. Moreover, SAHA treatment suppressed M1 cytokine expression (IL-6, TNF-α, and iNOS) while promoted the transcription of M2 cytokines (Arg-1 and IL-10) in LPS-challenged mouse microglia, and enhanced CD206 (M2 marker) but decreased CD86 (M1 markers) levels in microglia isolated from the ipsilateral hemisphere of MCAO mice. Collectively, our data suggested that the protection of SAHA on ischemic brain injury was closely associated with its inhibition on the early inflammatory response, and this inhibition was related to its reducing microglia activation and priming the activated microglia toward a more protective phenotype.

## Introduction

Ischemic stroke is a leading cause of death and adult disability worldwide (1); aside from tissue plasminogen activator (tPA), available treatments are minimal (2, 3). Protein acetylation modulated by histone acetyltransferases (HAT) and histone deacetylases (HDAC) is a widespread posttranslational modification (4, 5). In the past decade, multiple studies highlighted protein acetylation is essential for maintaining the homeostasis of the central nervous system, and its balance is frequently disrupted under disease and injury states, including stroke, and multiple neurodegenerative disorders (6, 7). Interestingly, accumulating *in vivo* and *in vitro* evidence proved modulation of protein acetylation by HDACi could mitigate ischemia-induced brain damage and promote endogenous regeneration and recovery (8, 9). They are therefore considered a promising therapeutic intervention for stroke and a variety of neurodegenerative diseases. Post-ischemic inflammation is a hallmark of ischemic stroke pathology, which plays critical roles in acute brain damage and profoundly affects long-term recovery (10, 11). Notably, it is a time-dependent process, starting with acute and intense inflammation and followed by a prolonged and mild one (12, 13). Microglia, the resident macrophages of the brain, and macrophages derived from infiltrated peripheral monocytes/macrophages are the two main elements participating in this immune response (14). These are two ontogenetically distinct cell populations and are activated or recruited with distinct kinetics after the onset of ischemia (14, 15).

Moreover, the roles of microglia and macrophages in the post-stroke inflammatory response are further complicated by their plasticity, as both of them can adopt different phenotypes in response to different extracellular milieu (16, 17). The two well-characterized are the “classically activated” M1 phenotype and the “alternatively activated” M2 phenotype. Generally, classically activated microglia/macrophages exert cytotoxic effects by releasing pro-inflammatory factors, which exacerbate brain infarction and damage (18). In contrast, alternatively activated microglia/macrophages exhibit an anti-inflammatory phenotype and promote brain recovery by clearing cell debris and releasing some anti-inflammatory cytokines and trophic factors. Accordingly, modulating the balance between the pro- and anti-inflammatory phenotypes represent a novel and promising strategy for stroke treatment (19).

To date, several studies reported the involvement of the anti-inflammatory effects of HDACi in their neuroprotection in acute brain ischemia (20, 21). In these studies, the long-term treatment of HDACi with multi-injection strategy was adopted. However, given the time-dependent inflammatory response of stroke (10) and the multipotency of HDACi (22), this long-term treatment is hard to figure out the mechanism underlying the anti-inflammatory effects of HDACi. Here, we proposed whether targeting the acute and intense early inflammatory responses by HDACi could achieve a protective effect on acute brain ischemia? More importantly, this single administration strategy could help us to understand the cellular mechanisms underlying the neuroprotection of HDACi.

To test the hypothesis, we compared the protection of a single dose of SAHA, an FDA-approved pan-HDACi, administrated at early and late time points in a tMCAO mouse model. Then, the anti-inflammatory effects of SAHA were determined by its impact on the expression of inflammatory cytokine, as well as microglia activation and the infiltration of peripheral monocytes. Finally, the impact of SAHA on microglia polarization was examined *in vitro* and *in vivo*. Our data showed the better protection of early SAHA treatment and suggested this protection was closely associated with its anti-inflammatory effect in the early stage of brain ischemia. Moreover, its anti-inflammatory effect was closely related to its reducing microglia activation and priming the activated microglia toward a more protective phenotype.

## Materials and Methods

### Animals and Ethics

Male C57BL/6 mice, 8–10 weeks old (22–26 g), were purchased from Guangdong medical laboratory animal center and adapted to the environment for 1 week before the experiments. CX3CR1^GFP/GFP^ mouse strains on a C57BL/6 background were obtained from the Jackson Laboratory. The experimental protocols and procedures involving animals and their care were conducted according to the National Institutes of Health Guide for Care and Use of Laboratory Animals and were approved by the Animal Experimental committee of Jinan University (20150302012).

### Animals Experimental Design

**Figure 1:**
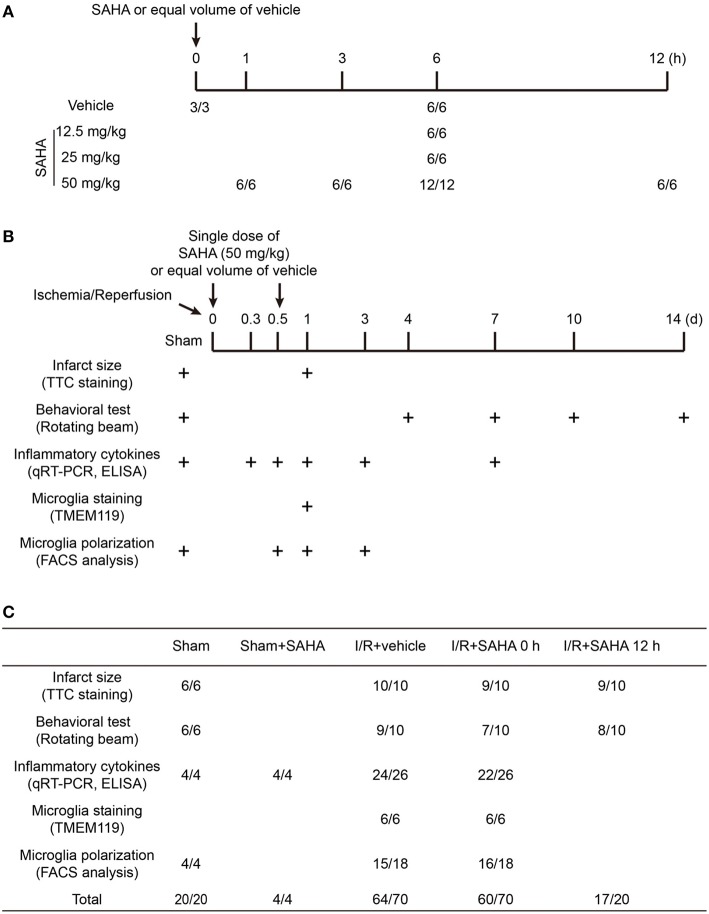
Schematic overview of the animal experimental design and group allocation. The timeline for determining the dosage and time-dependent effects of SAHA on protein acetylation in mouse brain **(A)**. The timeline for evaluating the protective effects of SAHA treatment on I/R **(B)** and the group allocation and number of mice in each experiment **(C)**. “+” represents the time for tissue sampling and behavior test. “*/*” represents the number of mice at the beginning and end of experiments. The difference was the number of mice died during the experiments.

In this study, animals were randomly allocated into three to five groups, and the number of animals in each group included in each analysis were illustrated in Figure 1: (1) Early SAHA treatment group (I/R + SAHA 0 h): SAHA (purity 99.77%, Selleck) was dissolved in a solution containing 25% dimethyl sulfoxide (DMSO) and 75% phosphate-buffered saline (PBS). SAHA (50 mg/kg) was intraperitoneally (i.p.) injected immediately after the onset of ischemia. (2) Late SAHA treatment group (I/R + SAHA 12 h), MACO mice were treated with SAHA (50 mg/kg) at 12 h after the onset of ischemia. (3) Vehicle group (I/R + Vehicle): MCAO mice were treated with an equal amount of vehicle (25% DMSO in PBS). (4) SAHA treated sham group, Sham-operated mice received SAHA (50 mg/kg) after surgery. (5) Sham group, Sham-operated mice received an equal amount of vehicle after surgery. At the indicated time point (Figure 1B), a behavioral test was performed, tissue samples were collected for TTC staining, RT-PCR, immunofluorescence staining, and FACS analysis. All the procedures in the behavioral test were carried out in a blinded fashion.

### Transient Middle Cerebral Artery Occlusion (tMCAO) Model

Anesthesia was induced with 4% isoflurane and maintained on 2% isoflurane in the air. Transient cerebral ischemia was caused by occlusion of the left middle cerebral artery with a monofilament suture (Doccol, 602356PK10) for 60 min. Rectal body temperature was maintained at 37 ± 0.5°C during surgery. Sham-operated mice were subjected to all surgical procedures except suture advancement. Neurological function was evaluated using a 0–5 point scale neurological score: 0 = no neurological dysfunction; 1 = failure to extend left forelimb fully when lifted by tail; 2 = circling to the contralateral side; 3 = falling to the left; 4 = no spontaneous walk or in a comatose state; 5 = death. Mice that exhibited the score of 0, 4, and 5 after surgery were excluded at this time. Animals were allotted randomly to the groups according to simple randomization. The surgeon was blinded to group allocations.

### Behavioral Test

Rotating beam test was adopted to evaluate the motor function as described (23). In brief, the rotating beam test measured the distance traveled and the speed of mice placed on a rotating beam (120 cm length, 15 mm diameter, and 3 rpm). The beam was located 60 cm above a table covered with a cushion to protect the mice from injury. Mice were pre-trained for 1 week before surgery, and each animal was given three trials per day.

### Western Blotting

Mice were euthanized by over anesthesia and transcardially perfused with ice-cold PBS. The Brain was dissected and sonicated in T-PER (tissue protein extraction reagents) (Pierce, USA) containing protease inhibitors (Complete C, Roche). The lysates were centrifuged at 12,000 g for 20 min at 4°C, and the supernatants were collected. After quantification by BCA protein assay (Pierce, USA), samples with equal protein were separated on 12% SDS-PAGE gels (30 μg protein per lane) and blotted onto PVDF (polyvinylidene difluoride) membrane (Millipore, Billerica, MA). According to the manufacturer's instruction, the blots were blocked with 1% BSA and incubated overnight at 4°C with antibodies against acetyl-histone H3 (Lys27) (1:1000, #4353, Cell Signaling Technology, Danvers, MA) or Acetylated-Lysine (1:1000, #9411, Cell Signaling Technology, Danvers, MA) in blocking buffer. After washing, the membranes were incubated with HRP-conjugated secondary antibodies for 1 h at room temperature. After another washing, ECL (Beyotime Biotechnology, China) was used to visualize the blotted proteins.

### TTC and Nissl Staining and Infarct Volume Measurement

For TTC (2, 3, 5-triphenyltetrazolium chloride) staining, mice brains were removed and sectioned into coronal slices (1 mm thickness) in a brain matrix (RWD Life Science, Shenzhen). The slices were immediately incubated in 1% TTC (Sigma-Aldrich, St. Louis, MO) in PBS for 30 min at 37°C and fixed with 4% paraformaldehyde (PFA) overnight. For Nissl staining, mice were anesthetized and transcardially perfused with 50 ml ice-cold PBS and 30 ml 4% paraformaldehyde (PFA). Dissected brains were post-fixed overnight in 4% PFA and followed by cryoprotection in 30% sucrose in PBS for 72 h. Coronal sections (40 μm thickness) were cut by a vibratome (Leica, Buffalo Grove, IL) and Nissl staining was performed on free-floating sections. The brain slices were then scanned, and infarction volume was measured by using ImageJ software (http://imagej.nih.gov/ij/). The indirect infarct area, in which the intact area of the ipsilateral hemisphere was subtracted from the area of the contralateral hemisphere, was calculated as described by Lin et al. (24).

### ELISA

Mice were sacrificed (*n* = 4–5 per group), and the whole brains were retrieved at 8 h after I/R. ELISA was performed following the protocol of the manufacturer to determine the concentration IL-1β, IL-6, and TNF-α (88-7013, 88-7064, 88-7324, Invitrogen) in the brain homogenates. Samples were run in triplicate, and all studies were performed blinded to treatment condition.

### Immunofluorescent Staining and Cell Counting

For immunostaining, the fixed brains were cut coronally at 50 μm thickness by a vibratome (Leica, Buffalo Grove, IL). Sections were permeabilized in 0.1% Triton X-100 in PBS for 10 min and incubated in blocking buffer (10% BSA in PBS) overnight at 4°C. Then, sections were incubated with the rabbit polyclonal anti-TMEM119 (1:200, 27585-1-AP, Proteintech), mouse monoclonal anti-PCNA (1:50, 60097-1-Ig, Proteintech), mouse monoclonal anti-IL-1β (1:200, sc-52012, Santa Cruz), rabbit monoclonal anti-Arg1 (1:500, 93668, Cell Signaling Technology), and Alexa Fluor 488 or 555-conjugated secondary antibodies (1:500, Cell Signaling Technology). Nuclei were counterstained with DAPI (1 μg/mL, Sigma). Fluorescence images were taken using a Zeiss LSM 700 confocal microscope (Carl Zeiss, Oberkochen, Germany). For cell counting, six ROIs (the region of interest) along the border of the ischemic ipsilateral hemisphere were counted. Data were expressed as the mean value of the cells in the ROIs. Cell counting was performed by blinded investigators.

### RNA Extraction and Quantitative Real-Time PCR Analysis

For RNA extraction, mice were anesthetized and transcardially perfused with 50 mL of ice-cold PBS. Total RNA was extracted from left hemisphere using Trizol reagent (life science). PrimeScript first strand cDNA Synthesis Kit (Takara Biotechnology, Dalian) was used to synthesize the first-strand cDNA from 1 μg total RNA. RT-PCR was performed on the Opticon 2 Real-Time PCR Detection System (Bio-Rad) using corresponding primers and SYBR gene PCR Master Mix (Invitrogen). The cycle time (Ct) value of the target gene was first normalized with that of β-actin from the same sample, and then the relative differences between control and treatment group were calculated and reported as fold changes compared to control. Primers were listed in Table 1.

**Table 1:** Primers for real-time polymerase chain reaction.

**Gene**	**Primer sequence**	
*Tnf-α*	Forward primer:	ATGGCCTCCCTCTCATCAGT
	Reverse primer:	TGGTTTGCTACGACGTGGG
*Il-1β*	Forward primer:	TGCCACCTTTTGACAGTGATG
	Reverse primer:	AAGGTCCACGGGAAAGACAC
*Il-6*	Forward primer:	GCCTTCTTGGGACTGATGCT
	Reverse primer:	TGCCATTGCACAACTCTTTTCT
*Arg-1*	Forward primer:	TTTTAGGGTTACGGCCGGTG
	Reverse primer:	CCTCGAGGCTGTCCTTTTGA
*Ym1*	Forward primer:	CAGGGTAATGAGTGGGTTGG
	Reverse primer:	CACGGCACCTCCTAAATTGT
*Il-10*	Forward primer:	CCAAGCCTTATCGGAAATGA
	Reverse primer:	TTTTCACAGGGGAGAAATCG
*Tgf-β*	Forward primer:	TGCGCTTGCAGAGATTAAAA
	Reverse primer:	CGTCAAAAGACAGCCACTCA
*Tlr4*	Forward primer:	GGCAACTTGGACCTGAGGAG
	Reverse primer:	CATGGGCTCTCGGTCCATAG
*β-actin*	Forward primer:	GGACTCCTATGTGGGTGACG
	Reverse primer:	CTTCTCCATGTCGTCCCAGT

### Brain Mononuclear Cells Isolation and Flow Cytometry Analysis

Brain mononuclear cells were collected as described (25). In brief, the brain was dissected after transcardial perfusion with 50 mL of cold PBS. Ipsilateral hemispheres were homogenized and filtered through a 70 μm cell strainer (BD Falcon 352350). After centrifugation at 1,400 rpm for 10 min, cell pellets were re-suspended in 37% Percoll (GE Healthcare, Pittsburg, PA) and 2 mL 70% Percoll was then carefully loaded under the cell suspension. After 30 min centrifugation at 2,000 rpm, cells at the interphase were collected and washed with 1% BSA in PBS. After resuspension in 200 μL staining buffer, an aliquoted cell was stained with trypan blue (Sigma-Aldrich, St. Louis, MO) and counted using a cell counter. Then cells were co-stained for 30 min on ice with PE Cy7-labeled anti-mouse CD45 (Biolegend, clone 30-F11), FITC-labeled anti-mouse CD11b (Biolegend, clone M1/70), Alexa Flour 647-labeled anti-mouse Ly6G (Biolegend, clone 1A8), Pacific Blue-labeled anti-mouse Ly6C (Biolegend, clone HK1.4), APC-labeled CD86 (Biolegend, clone FA-11) and CD206 (Biolegend, clone 15-2). Afterward, cells were washed with 1% BSA in PBS and filtered through a cell strainer cap (BD Falcon, REF 352235). Before analyzing on a FACSAria (BD Biosciences, USA), 2 μL Propidium Iodide (PI) (200 μg/mL, Sigma) was added, and 5,000 living cells were acquired. FACS data were analyzed by Flowjo (Treestar, USA).

### Primary Microglia Culture

Primary microglial cultures were prepared as described by Saura et al. (26) but with some modifications. Briefly, primary mixed glial cultures were prepared from brains of C57BL/6 newborn pups. After trypsin digestion (0.1% in PBS, Corning, China) and centrifugation at 400 g for 5 min, cells precipitated from four brains were inoculated into a 75 cm^2^ flask. When the cells were confluent, microglial cells were obtained by shaking the flasks. The detached cells were pelleted at 400 g for 5 min and inoculated at 20,000 cells/cm^2^ in 6 or 12-well plates pre-coated with 0.05% poly-D-lysine (Sigma-Aldrich, St. Louis, MO). Both primary and subculture of microglia were incubated at 37°C, 5% CO_2_ under saturating humidity.

### Cell Proliferation, Migration, and Apoptosis Assays

For cell proliferation assay, microglia cells were plated in 24-well plates at a density of 2 × 104 cells/well. Before the end of treatment, 10 μM EdU (RiboBio, Guangzhou, China) was added to the culture medium for 30 min. After fixation and washing, cells were stained according to the manufacturer's instructions. The Proliferation index (%)=EdU positive counts/counts of total cells × 100%.

Migration of microglia was measured by using a Transwell chamber (8 μm pore size, 6.5 mm diameter; Corning Life Sciences, Lowell, MA, USA). Briefly, 2 × 10^4^ microglia in completed medium (10% FBS DMEM) were seeded into the upper chamber of the insert. One hour later, the medium in the upper chamber was replaced by complete medium containing LPS (100 ng/ml) and SAHA (4 μM), and the lower chamber was replaced by completed medium containing 300 μM ATP (Sigma-Aldrich, St. Louis, MO). After another 23 h culture, cells were fixed with 4% paraformaldehyde and stained with 0.1% Crystal Violet (Beyotime technology). The migration rate was quantified by counting the migration cells in six random fields under a light microscope (Carl Zeiss, Oberkochen, Germany).

Apoptosis of microglia cells was determined using a Terminal deoxynucleotidyl transferase-mediated dUTP nick end labeling (TUNEL) detection kit (Roche, Penzberg, Germany). Microglia were seeded into 24-well plates containing cover glass at a density of 2 × 10^4^ cells/well. According to the manufacturer's protocol, microglia cells were fixed with 4% paraformaldehyde in PBS for 30 min at room temperature and permeabilized with 0.1% Triton X-100 in 0.1% sodium citrate for 2 min on ice. After washing in PBS, cells were incubated with the TUNEL reaction mixture for 1 h at 37°C. After washing, the stained cells were visualized using a fluorescence microscope (Carl Zeiss, Oberkochen, Germany).

### Statistically Analysis

All data were expressed as mean ± SEM, and statistical analysis was conducted by GraphPad Prism Software (GraphPad Software, LaJolla, CA). Two-way ANOVA repeated measures with *post hoc* Bonferroni comparison was used for time course studies of rotating beam and inflammatory cytokines. Comparisons between two groups and multiple groups were carried out by the Student's t-test and one-way ANOVA, respectively. Differences with *p* < 0.05 were considered statistically significant. Unless otherwise indicated, all experiments were repeated three times.

## Results

### SAHA Increases Protein Acetylation Levels in the Mouse Brain in a Time- and Dose-Dependent Manner

**Figure 2:**
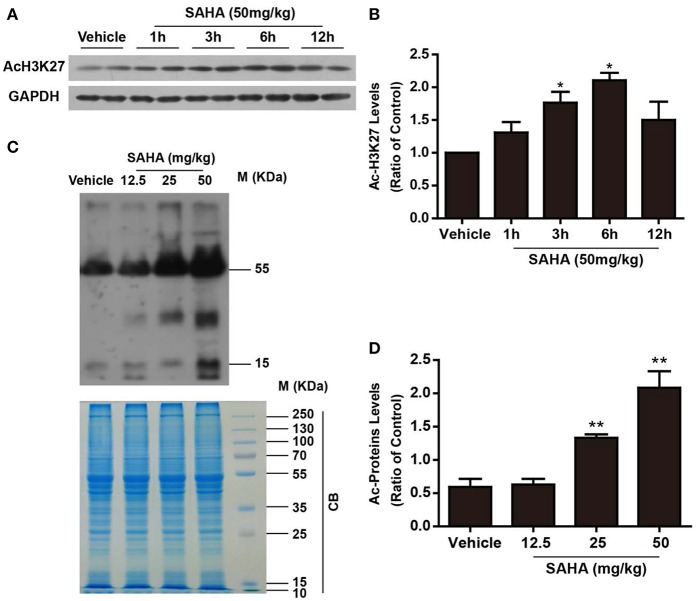
The time- and dose-dependent effects of SAHA on protein acetylation levels in normal mouse brain. **(A)** The time-dependent effects of SAHA on histone acetylation. Normal mice received a single dose of SAHA (50 mg/kg, i.p.) and were sacrificed 1, 3, 6, and 12 h after injection. Proteins separated via SDS-PAGE were detected using an antibody against acetylated lysine (K) 27 of histone H3 (AcH3K27). GAPDH was used as a loading control. **(B)** Densitometry analysis of acetylated H3K27, *p < 0.05, **p < 0.01 compared with vehicle control. **(C)** The dose-dependent effects of SAHA on global lysine acetylation of brain proteins. Normal mice treated with 12.5, 25, or 50 mg/kg SAHA were sacrificed at 6 h, and proteins extracted from brains were separated via SDS-PAGE and detected using an antibody against acetyl-lysine. Coomassie brilliant blue (CB) staining was used as a loading control. **(D)** Densitometry analysis of global lysine acetylation levels of mouse brain proteins. *p < 0.05; **p < 0.01 compared with vehicle control, *n* = 6/group.

To detect the time-dependent effects of SAHA on modulating protein acetylation in the mouse brain, normal mice were injected intraperitoneally (i.p.) with a single dose of 50 mg/kg SAHA and sacrificed at 1, 3, 6, and 12 h (Figure 1A). H3K27 acetylation, a well-known target of SAHA (27), was detected by western blotting. Compared with vehicle control, the levels of H3K27 acetylation in mouse brain began to increase 1 h after injection, reached a peak at 6 h and then declined (Figures 2A,B), indicating a relatively short active time of SAHA. A similar result was reported in human plasma, where the mean half-life (t1/2) of SAHA ranged from 91.6 to 127 min, and acetylated histone H3 accumulation was observed in peripheral blood mononuclear cells from 2 to 10 h, depending on the dose (28).

Then the dose effects of SAHA on the acetylation profile of brain were examined by using a pan-anti-acetyl-lysine antibody. Normal mice i.p. injected with a single dose of 12.5, 25, and 50 mg/kg SAHA or vehicle were sacrificed at 6 h after injection (Figure 1A). SAHA treatment increased global protein acetylation levels in a dose-dependent manner (Figures 2C,D), and 50 mg/kg SAHA was used in subsequent experiments. Notably, besides bands at ~15 kDa which corresponded to histones, other bands, especially those at ~30 and 55 kDa (Figure 2C), were intensively acetylated, suggesting the acetylation status of multiple non-histone proteins in the mouse brain were regulated by SAHA.

### Immediate SAHA Treatment Reduces Ischemia and Reperfusion (I/R) Brain Injury and Improves Functional Recovery

**Figure 3:**
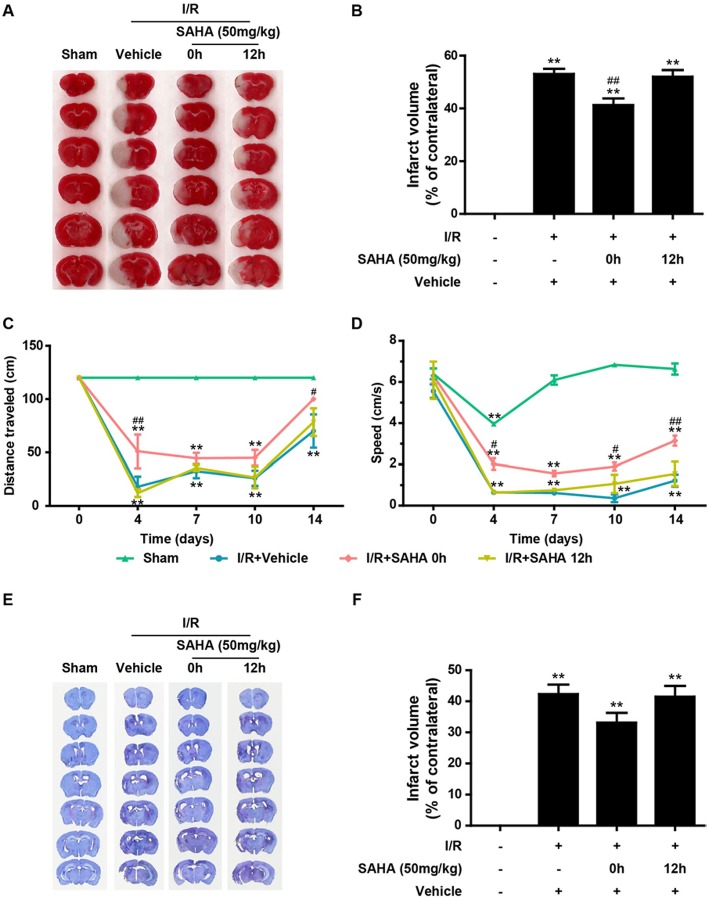
Immediate post-ischemic SAHA treatment reduces infarct volume and improves functional recovery in tMCAO mice. Mice subjected to 60 min of t-MCAO were injected with SAHA (50 mg/kg, i.p.) immediately or 12 h after ischemia onset. An equal volume of vehicle was injected as a control. Mice were sacrificed after 24 h, and TTC staining was performed to examine the infarct volume. **(A)** A representative diagram showing the brain infarction detected using TTC staining in six coronal brain sections from sham, vehicle, and two SAHA treatment groups. **(B)** Infarct volumes quantified by the TTC staining. **p < 0.01 vs. sham control; ^*##*^*p* < 0.01 vs. vehicle control, *n* = 6–10/group. Effects of SAHA treatment on speed **(C)** and distance **(D)** were measured before surgery and 4–14 days after the onset of I/R. **p < 0.01 compared with time point 0, ^#^*p* < 0.05; ^##^*p* < 0.01 compared with I/R+vehicle group, *n* = 6–10/group. **(E)** Nissl staining of seven coronal brain sections from the sham, vehicle, and two SAHA treatment groups. **(F)** Infarct volumes quantified by the Nissl staining. **p < 0.01 compared with the sham group, **p < 0.01 compared with I/R+vehicle group, *n* = 6–10/group.

To determine whether target the early inflammatory response could achieve the protection of HDACi, mice subjected to tMCAO for 60 min were treated with a single dose of SAHA (50 mg/kg) either immediately or 12 h after the onset of ischemia. The infarct volume was measured by TTC staining at 24 h after ischemia onset (Figures 1B,C). I/R produced an infarct volume of ~55%, and immediate SAHA treatment significantly reduced the infarct volume by around 25%, compared with that of the vehicle control group (Figures 3A,B, *p* < 0.01). A smaller infarct volume was also observed in the delayed treatment group, though the difference was not statistically significant (Figures 3A,B, Supplemental Table 1).

To evaluate the influence of SAHA injected at different times on the recovery of neurological function, the rotating beam test, a relatively sensitive sensory-motor behavior test (23), was performed as described in the Methods. I/R brain injury induced a dramatic deterioration of neurological function, indicated by the significant reduction in the speed and distance of mice subjected to tMCAO (Figures 3C,D). Compared with the vehicle control, immediate SAHA treatment markedly improved the distance (Figure 3C) and speed (Figure 3D) of tMCAO mice, while delayed SAHA treatment showed little improvement (Figures 3C,D), highlighting the time-dependence of SAHA-mediated protection.

At the end of the behavior test, all the animals were sacrificed, and Nissle staining was performed to determine the long term effects of SAHA on lesion volume. The mean lesion volume of 0 h SAHA treatment group was smaller than that of the vehicle control and 12 h treatment, but there is no statistically significant difference among these three groups (Figures 3E,F).

### Early SAHA Treatment Suppresses the Overexpression of Pro-inflammatory Cytokines

**Figure 4:**
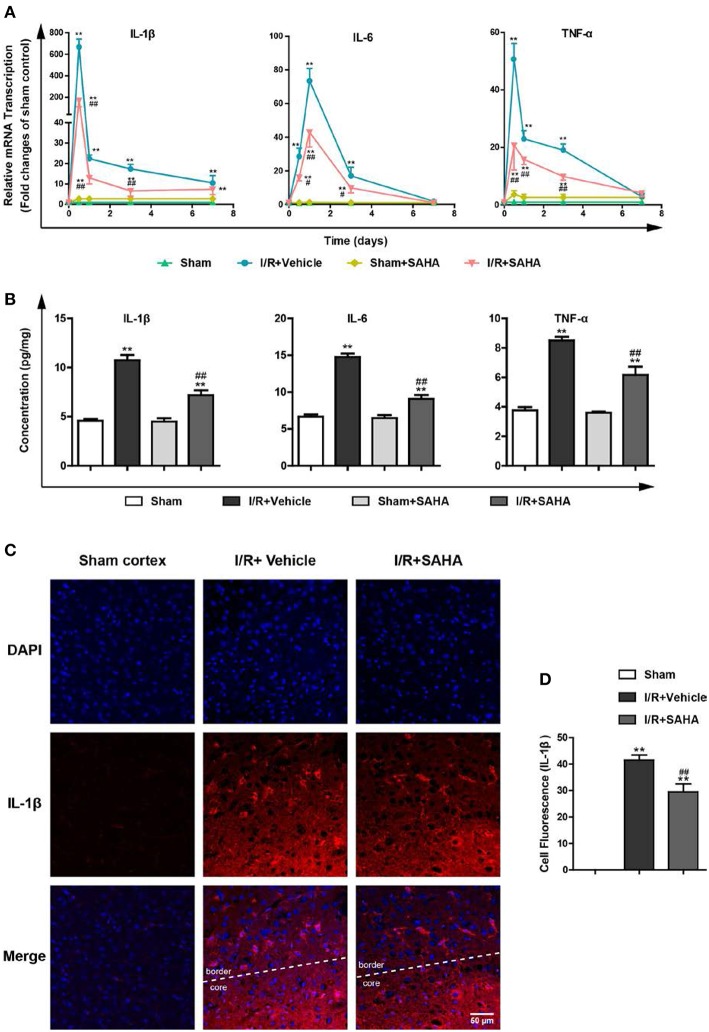
SAHA suppresses the overexpression of pro-inflammatory cytokines induced by I/R. Mice subjected to 60 min of t-MCAO were injected with SAHA (50 mg/kg, i.p.) immediately after ischemia onset. An equal volume of vehicle was injected as a control. Mice were sacrificed at 8 h, and on day 1, 3, and 7 after I/R. **(A)** The transcription of IL-1β, IL-6, and TNF-α in the ipsilateral hemisphere was examined by RT-PCR. **(B)** The concentration of IL-1β, IL-6, and TNF-α was detected by ELISA in the whole brain at 8 h after I/R. **(C)** Immunofluorescent staining showed IL-1β positive cells were mainly distributed in the border of the ipsilateral hemisphere on day 1 after I/R. Scale bar, 50 μm. **(D)** Quantification of the Fluorescence Intensity of IL-1β positive cells. **p < 0.01 compared with the Sham group. ^##^*p* < 0.01, compared with I/R+Vehicle group, *n* = 4–6/group.

I/R induces the excessive production of pro-inflammatory cytokines, such as IL-1β, IL-6, and TNF-α, which exacerbate ischemic brain injury (13, 29). To explore the anti-inflammatory properties of SAHA, we examined the effects of SAHA treatment on the temporal expression of these pro-inflammatory cytokines in the ipsilateral hemisphere of MCAO mice using RT-PCR. As reported (13), I/R injury induced a rapid increase in pro-inflammatory cytokine levels, transcripts of these three cytokines peaked at 8 h after I/R, then declined and returned to baseline on day 7. Immediate SAHA treatment significantly suppressed the transcripts of all these three cytokines from 8 h to day 3 (Figure 4A). Then, ELISA assay was performed to measure the effects of SAHA on the concentration of these cytokines in the whole brain at 8 h after I/R. Consistently, all three cytokines induced by ischemia were suppressed by SAHA (Figure 4B). Further immunofluorescent staining showed IL-1β positive cells mainly distributed in the border of the ipsilateral hemisphere (Figure 4C). Again, SAHA treatment significantly decreased the MFI of IL-1β positive cells (Figure 4D).

### SAHA Decreases the Number of Activated Microglia and Delays the Infiltration of Monocytes

**Figure 5:**
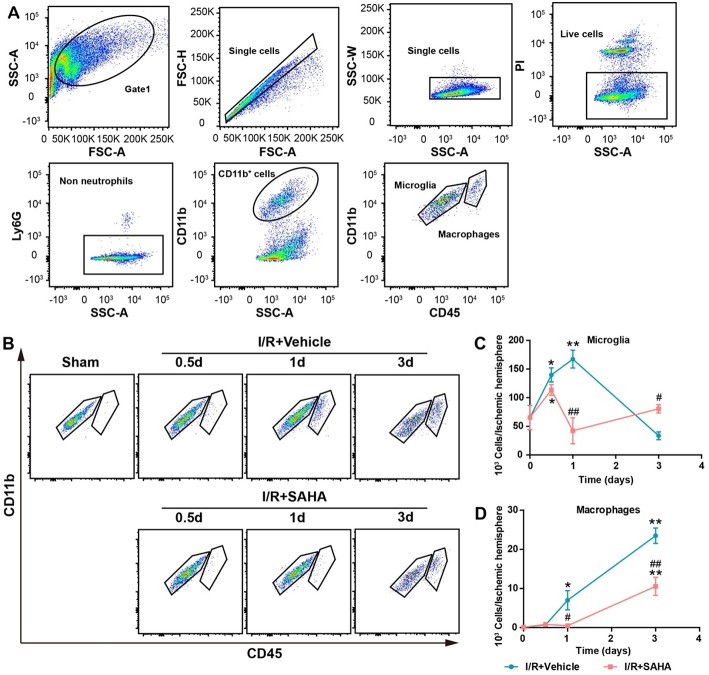
Effects of SAHA treatment on microglia activation and macrophage infiltration in the ipsilateral hemisphere of tMCAO mice. **(A)** Representative dot plots depicting the gating strategy of neutrophils (Ly6G^+^), resident microglia (Ly6G^−^CD11b^+^CD45^medium^), and macrophages (Ly6G^−^CD45^high^CD11b^+^) from the mononuclear cells isolated from the ipsilateral hemispheres of 3 d tMCAO mouse. Dead cells were first excluded from single cells. **(B)** Representative dot plots showing the effects of SAHA treatment on the temporal changes of microglia and macrophages at 0.5, 1, and 3 d after the onset of brain ischemia. The temporal dynamics of microglia **(C)**, macrophages **(D)** at 0.5, 1, and 3 d after I/R. *p < 0.05; **p < 0.01, compared with Sham group. ^#^*p* < 0.05; ^##^*p* < 0.01 compared with I/R + vehicle group. *n* = 4–6/group/time point.

During the post-stroke inflammatory response, microglia activation, and the infiltrated monocytes/macrophages are a time-dependent course (14). FACS analysis was performed to further discriminate the effects of SAHA treatment on resident microglia activation and peripheral leukocyte infiltration. In this study, a modified gating strategy was established to exclude potential interference from dead cells, which is a frequently overlooked issue in these studies (30, 31). First, dead cells were stained using PI and excluded from single cells; then neutrophils were gated on Ly6G^+^ staining. Finally, CD45 was used to discriminate resident microglia (Ly6G^−^/CD11b^+^/CD45^medium^) from infiltrated monocytes (Ly6G^−^/CD11b^+^/CD45^high^) (Figure 5A).

Mononuclear cells isolated from the ipsilateral hemisphere of tMCAO mice were analyzed and counted at 0.5, 1, and 3 d after I/R onset (Figures 5B,C). The number of microglia significantly increased on 0.5 d and peaked on day 1 in the vehicle control, but decreased to ~50% of that in the sham control on day 3 (*p* < 0.05, Figures 5B,C). Marked monocyte infiltration occurred from day 1, and this increasing trend maintained until day 3, when the number of infiltrated macrophages approximately equaled to that of microglia (Figures 5B–D). This temporal pattern is generally consistent with reports using the same mouse model (13).

Early SAHA treatment significantly altered the temporal dynamics of microglia activation and monocytes/macrophages infiltration (Figures 5B–D). SAHA reduced the number of microglia on day 1 (*p* < 0.05, Figure 5C), and markedly decreased the number of infiltrated monocytes/macrophages on day 1 to 3 (*p* < 0.05 or < 0.01, Figure 5D). Considering the relatively short active time of SAHA (Figures 2A,B) and the predominance of microglia rather than infiltrated monocytes/macrophages in the early stage of I/R brain injury (Figure 5), these results suggested microglia could be one of the targets of early SAHA treatment, and the protection of SAHA could be closely associated with the suppression of microglia activation in the early stage of I/R injury.

### SAHA Inhibits the Proliferation of Microglia in the MCAO Mice

**Figure 6:**
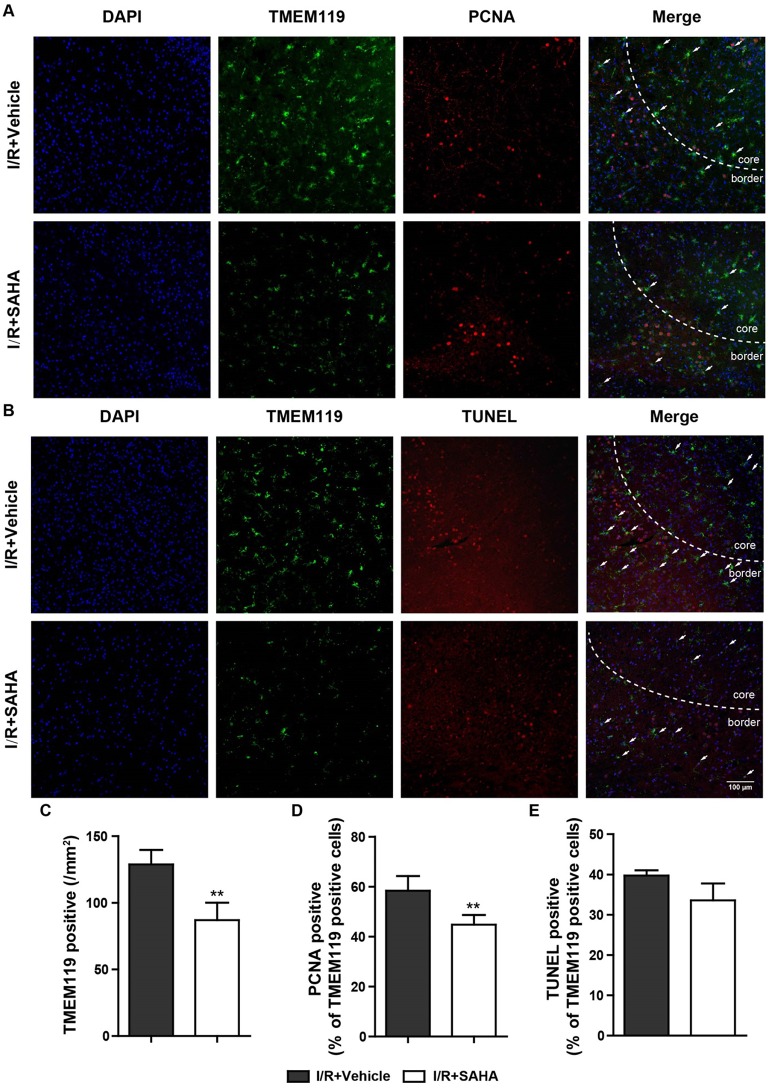
SAHA decreases the proliferation of microglia in MCAO mice. Representative double-staining images of TMEM119 and PCNA **(A)**, and TMEM119 and TUNEL **(B)** in the border of tMCAO mice at 24 h after I/R. Scale bar, 100 μm. **(C)** Quantification of the number of TMEM-119 positive cells, percentage of PCNA and TMEM119 double positive cells **(D)**, and TMEM119 and TUNEL double positive cells **(E)**. **p < 0.01 compared with vehicle control, *n* = 6/group.

To confirm SAHA treatment could reduce the number of microglia and explore the mechanism responsible for this reduction, tMCAO mice treated with or without SAHA immediately after the onset of ischemia were sacrificed at 24 h after I/R, and TMEM119, a marker for microglia (32), were co-stained with PCNA and TUNEL (Figure 6). Consistent with FACS data, the number of TMEM119 positive cells significantly decreased in SAHA group, which was around 65% of that in the vehicle control (Figures 6A–C). Moreover, SAHA treatment reduced the intensity of TMEM119 staining in the ipsilateral hemisphere (Figures 6A,B). As presented in Figures 6A,B, both PCNA and TUNEL positive cells mainly distributed in the infarct boundary, and some of them co-localized with the TMEM119 labels. Quantified results showed that SAHA treatment significantly declined the percentage of TMEM119 and PCNA double positive cells, but not TMEM119 and TUNEL double positive cells.

### SAHA Suppresses Mouse Microglia Proliferation and Migration but Not Apoptosis *in vitro*

**Figure 7:**
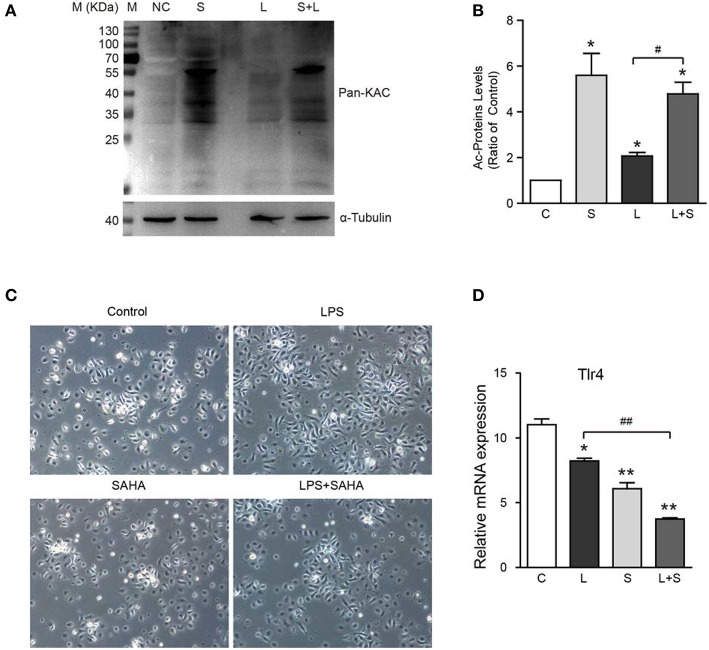
Effects of SAHA treatment on protein acetylation, cell morphology, and Tlr4 expression of mouse microglia. Mouse microglia were treated with 100 ng/ml LPS, 4 μM SAHA, 100 ng/ml LPS+4 μM SAHA for 12 h. Effects of treatment on protein acetylation **(A)**, cell morphology **(C)**, and transcription of Tlr4 **(D)** of mouse microglia were detected. **(B)** Quantification of the global lysine acetylation levels in **(A)**. *p < 0.05; **p < 0.01 compared with normal control. ^#^*p* < 0.05; ^##^*p* < 0.01 compared with LPS group. Scale bar, 50 μm, *n* = 3/group.

To further verify the inhibition of SAHA on microglia activation, 4 μM SAHA was added to mouse microglia exposed to 100 ng/mL bacterial lipopolysaccharide (LPS), a classical *in vitro* microglia activation model. SAHA significantly increased the global protein acetylation in normal and LPS activated mouse microglia (Figures 7A,B). LPS treatment resulted in a larger and longer cell body of microglia, an activated phenotype (Figure 7C). Notably, although SAHA alone did not change the morphology of microglia, it can reverse the effect of LPS (Figure 7C). Toll-like receptor 4 (Tlr4) is the canonical receptor for LPS. As showed in Figure 7B, LPS suppressed the transcription of Tlr4, and SAHA treatment strengthened this suppression (Figure 7D), implicating the involvement of Tlr4 pathway in suppressing microglia activation by SAHA.

**Figure 8:**
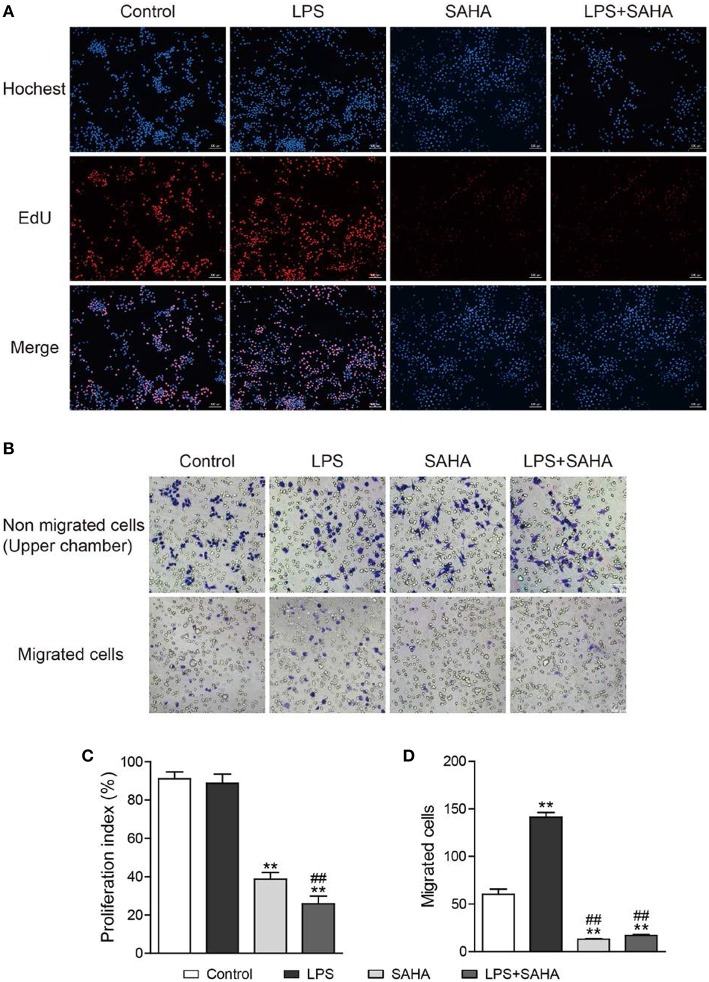
SAHA inhibits microglia proliferation and migration *in vitro*. Mouse microglia were treated with 100 ng/ml LPS, 4 μM SAHA, 100 ng/ml LPS+4 μM SAHA for 12 h. The effects of SAHA treatment on the proliferation and migration of mouse microglia were detected by EdU **(A)** and Transwell **(B)** assays, respectively. Quantitation of EdU-positive cells **(C)** and migrated cell **(D)** revealed SAHA treatment significantly inhibited the proliferation and migration of mouse microglia. **p < 0.001 compared with normal control; ^##^*p* < 0.01 compared with LPS group. *n* = 3/group.

**Figure 9:**
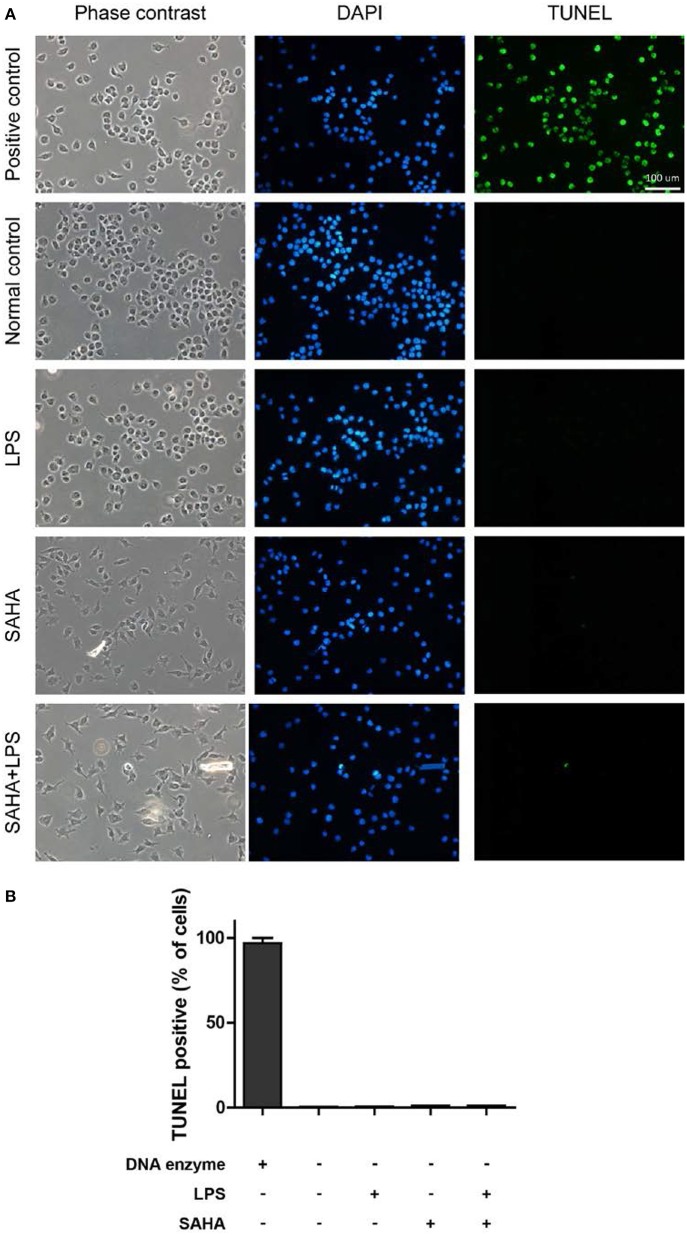
SAHA treatment does not induce mouse microglial apoptosis. **(A)** Mouse microglia were treated with 100 ng/ml LPS, 4 μM SAHA, 100 ng/ml LPS+4 μM SAHA for 12 h. The apoptosis was detected by TUNEL staining. The positive control is the same cells treated by DNase I. Scale bar, 100 μm. **(B)** Quantitation of the TUNEL-positive cells. No statistical significance between the treatment groups and normal control.

Then, EdU and Transwell assays were performed to explore the effects of SAHA treatment on cell proliferation and migration of microglia *in vitro*. In EdU assays, most microglia of control and LPS group were in the S phase of cell cycle and with similar proliferation index. In both cases, SAHA treatment significantly inhibited microglia proliferation (Figures 8A,C). Compared with the control group, more LPS activated microglia migrated through the Transwell insert membrane. Again, SAHA treatment suppressed not only the migration of normal cultured microglia but also those activated by LPS (Figures 8B,D). Consistent with the *in vivo* TUNEL result (Figures 6B,E), short term SAHA treatment did not produce visible apoptosis in microglia (Figures 9A,B). Therefore, these *in vivo* and *in vitro* results suggested that the reduction of microglia by SAHA treatment was closely associated with its inhibition on microglia proliferation and migration.

### SAHA Primes Activated Microglia Toward a More Protective Phenotype

**Figure 10:**
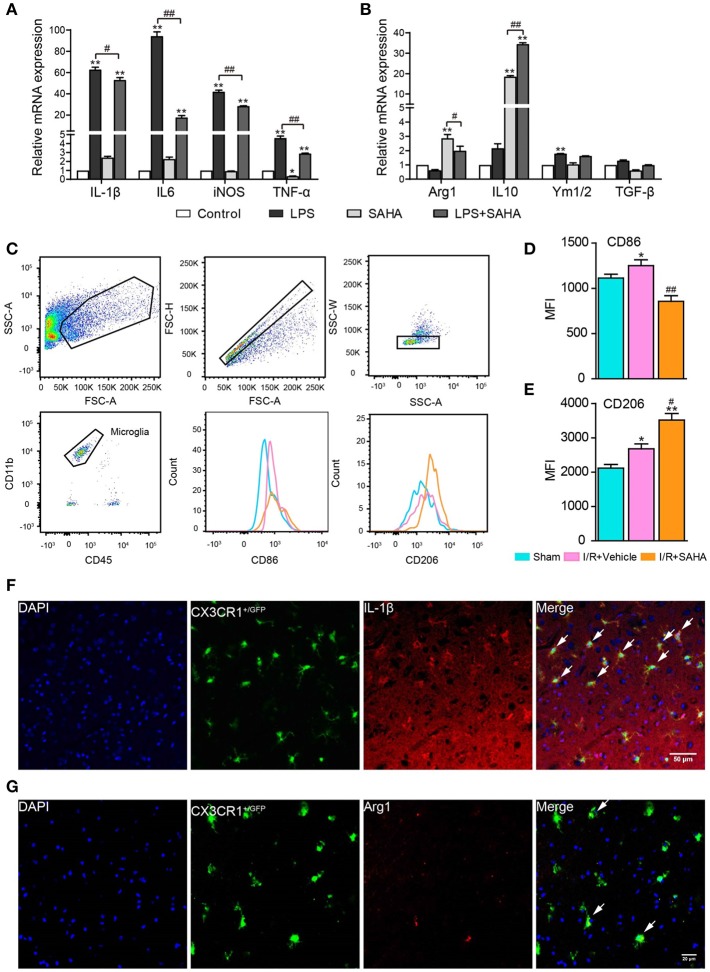
SAHA treatment primes activated microglia toward an M2 phenotype *in vitro and in vivo*. Effects of SAHA on the transcripts of pro-inflammatory cytokines (IL-6, IL-1β, TNF-α, and iNOS) **(A)** and anti-inflammatory cytokines (Arg1, Ym1/2, IL-10, and TGF-β) **(B)** in mouse microglia were detected using RT-PCR. Data are expressed as fold-change vs. the control group. *p < 0.05, **p < 0.01 compared with normal control; ^#^*p* < 0.05; ^##^*p* < 0.01 compared with LPS group. **(C)** Diagram depicting the gating strategy of microglia (CD11b^+^CD45^medium^). The histogram illustrates the effects of early SAHA treatment on the mean fluorescence intensity (MFI) of CD86 **(D)** and CD206 **(E)** in the microglia isolated from the ipsilateral hemisphere on day 1 after I/R, *n* = 4–6/group. Bar graph showing the MFI of CD86 (D) and CD206 (E). *p < 0.05, **p < 0.01 compared with Sham control; ^#^*p* < 0.05; ^##^*p* < 0.01 compared with I/R+vehicle. Immunofluorescent staining showed the co-localization of IL-1β **(F)** and Arg1 **(G)** with microglia (CX3CR1^+/GFP^) on day 1 after I/R. Arrows identify co-localized expression cells. Scale bar, 50 and 25 μm for IL-1β and Arg1, respectively.

Microglia in the central nervous system change their phenotypes in response to different microenvironments (17). To test the impact of SAHA treatment on the phenotypes of activated microglia, the expression of a panel of pro-inflammatory cytokines (IL-1β, IL-6, TNF-α, and iNOS) and anti-inflammatory cytokines (IL-10, YM1, TGF-β, and Arg-1) was examined. As expected, LPS treatment significantly and intensively stimulated the expression of all the examined pro-inflammatory cytokines, especially IL-6, IL-1β, and iNOS (*p* < 0.01, (Figure 10A). LPS treatment also slightly increased the transcription of anti-inflammatory cytokines YM1 (*p* < 0.05, Figure 10B). Notably, SAHA alone markedly suppressed TNF-α expression but improved IL-10 and Arg-1 expression (*p* < 0.01, Figures 10A,B), reflecting its inherent anti-inflammatory properties (33). More importantly, SAHA markedly suppressed overexpression of these pro-inflammatory cytokines stimulated by LPS (*p* < 0.01, Figure 10A), particularly IL-6, a cytokine predominantly expressed by microglia *in vivo*. At the same time, SAHA treatment increased Arg1 transcription and further enhanced IL-10 expression but had no significant effect on YM1 or TGF-β, similar to the effects of SAHA alone (Figure 10B).

To further characterize the modulatory effects of SAHA on microglia phenotype *in vivo*, FACS analysis was performed to assess the treatment on the expression of M1 and M2 phenotypic markers CD86 and CD206 in microglia isolated from the ipsilateral hemisphere. In this experiment, the dead cell exclusion step was removed because dead cells were nonspecifically stained as a CD45^+^/CD11b^high^ population and had little influence on the microglia population (Figure 10C). In this study, mononuclear cells isolated from the ipsilateral hemisphere of tMCAO mice treated with SAHA or vehicle for 24 h were analyzed. I/R injury increased the mean fluorescence intensities MFI of CD86 and CD206 in activated microglia (Figure 10D), but SAHA markedly decreased the MFI of CD86 while increased mean fluorescence intensity (MFI) of CD206 (Figure 10E). Immunostaining validated the colocalization of M1(IL-1β) and M2 (Arg1) markers with microglia (CX3CR1^GFP/−^) in MCAO mice (Figures 10F,G). Together, both *in vitro* and *in vivo* data indicated that SAHA treatment could prime activated microglia toward an M2 phenotype, a more protective phenotype.

## Discussion

In this study, we demonstrated that a single dose of SAHA treatment immediate after ischemia provided more protection than that of the delayed administration. This protection was closely associated with the inhibition of early inflammatory response, as supported by the significantly suppressed pro-inflammatory cytokines (IL-1β, IL-6, and TNF-α) (Figure 4). FACS analysis indicated SAHA treatment reduced the number of activated microglia in the early phase of post-stroke inflammation when the infiltration of peripheral monocytes/macrophages just began (Figure 5). Moreover, both *in vitro* and *in vivo* experiments showed SAHA treatment suppressed microglia proliferation (Figures 6, 8) and primed activated microglia toward M2 phenotype (Figure 10), a protective phenotype. In summary, our data suggested that the protective effects of SAHA on ischemic brain injury were closely associated with its inhibition on the early inflammatory response, and this inhibition was closely associated with reducing microglia activation and priming these activated microglia toward a more protective phenotype.

Acute I/R brain injury is followed by a robust and acute inflammatory response that is featured by the overexpression of pro-inflammatory cytokines, such as TNF-α, IL-1β, and IL-6 (13). These excessive inflammatory cytokines aggravate the ischemic brain injury and are closely related to poor outcomes in both animal models and stroke patients; nevertheless, inhibition of the inflammatory response mitigates ischemic damage and can benefit functional recovery (13, 34). Our data showed immediate SAHA treatment after ischemia significantly suppressed the rapid up-regulation and provided more protection than that of delayed administration (Figure 3), highlighting the importance of targeting the early inflammatory response to get the full protection of HDACi. Similarly, Kim et al. reported that pan-HDAC inhibitors sodium butyrate (SB) decreased stroke-induced infarct volume and reduce inflammation when administered within 3 h after stroke induction and the lower protection of SB injected at 6 h after ischemia onset (21). Another supporting evidence came from the multiple administration strategies adopted by almost all the studies exploring the protective effects of HDACi, in these studies, the first injection was performed immediately after the onset of ischemia (35, 36). However, several reports described the late administration of HDACi could also exert protective effects (37). This discrepancy seemed to reflect the multiple effects of HDACi on a different aspect of post-ischemic brain damage and recovery (22). On another hand, as the involvement of inflammation in multiple aspects of post-ischemic injury and recovery, such as BBB damage (38) and neurogenesis (39), the anti-inflammatory features of HDACi were likely responsible for their multiple protective effects.

Microglia are in the first-line in responding to ischemic brain injury, which becomes activation soon after brain ischemia (40). Activated microglia are characterized by cell proliferation and excessive production of pro-inflammatory cytokine (13). Consistent with this idea, the dramatic enhancement of pro-inflammatory cytokines in the early phase of I/R brain injury was paralleled with the increasing number of microglia in the ipsilateral hemisphere, notably, both of them peaked at 1 d, when the infiltration of macrophages and neutrophils just began (Figure 5). This temporal pattern indicated activated microglia were the primary sources of these detrimental pro-inflammatory cytokines in the early phase of I/R injury. In support of this notion, both Ritzel et al. (25) and Lambersten et al. (13) studies reported that resident microglia adopted a mostly pro-inflammatory phenotype in the early phase of MCAO, while macrophages and neutrophils did not play significant roles in the progression of ischemic neuronal damage up to 16 h (41). Considering the significantly decreased number of activated microglia by early SAHA treatment (Figures 5, 6), it was rational to postulate that microglia were the primary targets of early SAHA treatment in the acute phase of post-ischemic inflammation, and inhibiting microglia activation in the early phase of I/R brain injury could be an important cellular mechanism for the protective effects of HDAC inhibition.

Macrophages derived from the infiltrated monocytes are essential elements of the innate immune system and participate in almost all inflammatory responses. Our data showed early SAHA treatment markedly delayed the entry of peripheral monocytes and decreased the number of infiltrated monocytes (Figure 5). Since macrophages normally do not proliferate after they are recruited (42), the augmented numbers of macrophages mostly result from the recruitment of peripheral monocytes/macrophages. Peripheral monocytes are primarily recruited into infarct areas by the overexpression of pro-inflammatory cytokines (TNF-α, IL-6, and IL-1β) and the subsequent up-regulation of adhesion molecules in the injured brain (11). In this case, the delay and reduction in the recruitment of macrophages partially resulted from the suppressed microglia activation following SAHA treatment. However, it worthy noted that the involvement of other cell types in this reduction could not be ignored, such as vascular endothelial, astrocytes and neurons, and monocytes and neutrophils *per se*, because the phenotypes of all these cells can be modulated by HDACi (22), and sequentially and indirectly affect the activation and phenotype of microglia.

One interesting finding of this study was SAHA treatment primed activated microglia (M1 phenotype) toward a more protective M2 phenotype, as evidenced by the suppression of pro-inflammatory cytokines and simultaneous enhancement of anti-inflammatory cytokines, as well as the altered expression of phenotypic M1 and M2 markers (Figure 10). Modulating the phenotypes of activated microglia represents a promising strategy for the treatment of stroke and other inflammation-related neurological diseases (17, 43). Brogdon et al. were the first to report the specificity of HDAC inhibition in modulating innate and adaptive immune responses (44). They found LAQ824, a small-molecule HDACi, specifically inhibited Th1 effector cell activation and migration, but not Th2 effector cell, and proposed the potential application of HDACi to regulate the Th1 and Th2 balance in clinical settings (44). Our data suggested that priming the activated microglia toward an M2 phenotype could be another mechanism for the protective effects of post-stroke SAHA treatment. In support of this idea, the latest study by Patnala et al. also showed SB epigenetically regulated microglia from pro-inflammatory to anti-inflammatory phenotype during the ischemic stroke (45).

Then the next question is how these seemingly non-specific HDAC inhibitors possess relatively specific biological functions? Besides the well-studied epigenetic modulation, recent mass spectrometry (MS)-based proteomic studies disclosed thousands of cytoplasmic, mitochondrial and nuclear proteins were subject to acetylation (46). These proteins participated in multiple cellular processes, including metabolism, signaling, and immunity, such as NF-κB, a key transcription factor in the immune response (47). Recently, Durham et al. claimed that the HDACs promoted the inflammatory response by regulating the acetylation levels of a non-histone protein rather than increasing levels of gene expression as a result of increased histone acetylation (48). As both SAHA and LPS treatment significantly altered protein acetylation profile in mouse microglia (Figure 7C), further studies are warranted to explore how can SAHA or other HDACi suppress inflammatory response by modulating non-histone protein acetylation in microglia.

## Conclusions

This study suggested that the protection of immediate SAHA treatment on ischemic brain injury was closely associated with its inhibition on the early inflammatory response, and this inhibition was closely related to its reducing microglia activation and priming these activated microglia toward an M2 phenotype.

## Ethics Statement

All experimental protocols and procedures involving the use of animals and their care were conducted according to the National Institutes of Health Guide for Care and Use of Laboratory Animals and were approved by the Animal Experimental committee of Jinan University.

## Author Contributions

YJ and QJ conceived and supervised the study. SL, QS, XH, and XL performed experiments, collected the data, and participated in the data analysis. YJ, QJ, and SL prepared the figures and drafted the manuscript for publication. ZJ, XZ, and ZC contributed to the animal model and relevant experiments. HZ and JJ reviewed and revised the manuscript. All authors read and approved the submitted version of the manuscript.

### Conflict of Interest Statement

The authors declare that the research was conducted in the absence of any commercial or financial relationships that could be construed as a potential conflict of interest.

## Abbreviations

Arg-1: Arginase1
BCA: Bicinchoninic acid
BSA: Bovine serum albumin
CB: Coomassie brilliant
dMCAO: distal middle cerebral artery occlusion mouse model
DMSO: Dimethyl sulfoxide
ECL: Enhanced chemiluminescence
FACS: Flow cytometry
HAT: Histone acetyltransferases
HDAC: Histone deacetylases
i. p.: Intraperitoneal injection
I/R: Ischemic/reperfusion
IL-10: Interleukin-10
IL-1β: Interleukin-1β
IL-6: Interleukin-6
LPS: Lipopolysaccharide
MFIs: Mean fluorescence intensities
MS: Mass spectrometry
NF-κB: Nuclear factor kappa-B
NO: Nitric oxide
PBMNCs: Peripheral blood mononuclear cells
PBS: Phosphate-buffered saline
PFA: Paraformaldehyde
PVDF: Polyvinylidene difluoride
RT-PCR: Real-Time Polymerase Chain Reaction
SAHA: Suberoylanilide hydroxamic acid
SB: Sodium butyrate
SDS-PAGE: Sodium dodecyl sulfate-polyacrylamide gel electrophoresis
tMCAO: transient middle cerebral artery occlusion
TNF-α: Tumor necrosis factor-α
tPA: tissue plasminogen activator
T-PER: Tissue protein extraction reagents
TTC: 2,3,5-Triphenyltetrazolium chloride
YM1: Chitinase-like protein 3.
