# Supplementary figures and images for: Early Histone Deacetylase Inhibition Mitigates Ischemia/Reperfusion Brain Injury by Reducing Microglia Activation and Modulating Their Phenotype
Source: Front Neurol. 2019 Aug 20;10:893. doi: 10.3389/fneur.2019.00893 (PMC6710990; doi:10.3389/fneur.2019.00893)

Supplemental table 1. Infarct volume of the individual mouse (TTC staining)

**
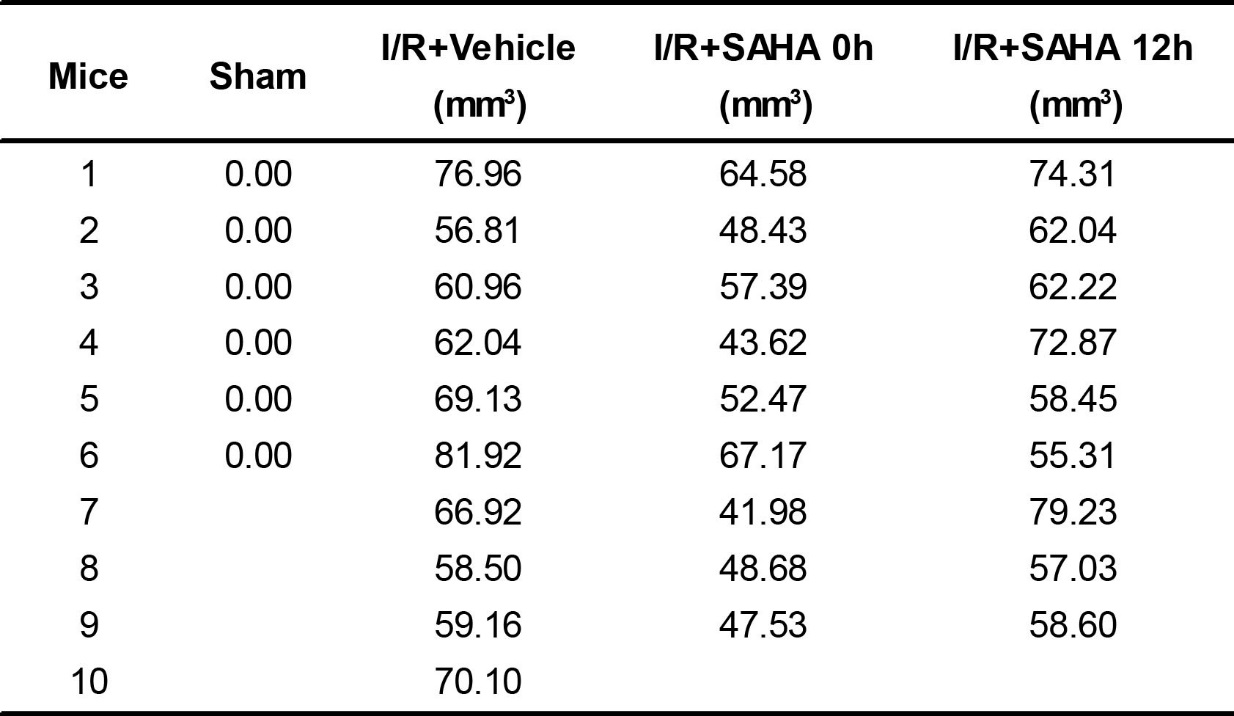
**

Supplement: Supplementary file 1 [file Data_Sheet_1.docx]
